# Etoposide induces cell death via mitochondrial-dependent actions of p53

**DOI:** 10.1186/s12935-015-0231-z

**Published:** 2015-08-07

**Authors:** Sarwat Jamil, Irene Lam, Maryam Majd, Shu-Huei Tsai, Vincent Duronio

**Affiliations:** Department of Medicine, Jack Bell Research Centre, Vancouver Coastal Health Research Institute, University of British Columbia, 2660 Oak St., Vancouver, BC V6H 3Z6 Canada

**Keywords:** Transcription, Mitochondria, DNA damage, Fibroblast, P53 acetylation

## Abstract

**Background:**

Etoposide has been used clinically in cancer treatment, as well as in numerous research studies, for many years. However, there is incomplete information about its exact mechanism of action in induction of cell death.

**Methods:**

Etoposide was compared at various concentrations to characterize the mechanisms by which it induces cell death. We investigated its effects on mouse embryonic fibroblasts (MEFs) and focused on both transcriptional and non-transcriptional responses of p53.

**Results:**

Here we demonstrate that treatment of MEFs with higher concentrations of etoposide induce apoptosis and activate the transcription-dependent functions of p53. Interestingly, lower concentrations of etoposide also induced apoptosis, but without any evidence of p53-dependent transcription up-regulation. Treatment of MEFs with an inhibitor of p53, Pifithrin-α, blocked p53-dependent transcription but failed to rescue the cells from etoposide-induced apoptosis. Treatment with PES, which inhibits the mitochondrial arm of the p53 pathway inhibited etoposide-induced cell death at all concentrations tested.

**Conclusions:**

We have demonstrated that transcriptional functions of p53 are dispensable for etoposide-induced cell death. The more recently characterized effects of p53 at the mitochondria, likely involving its interactions with BCL-2 family members, are thus more important for etoposide’s actions.

**Electronic supplementary material:**

The online version of this article (doi:10.1186/s12935-015-0231-z) contains supplementary material, which is available to authorized users.

## Background

p53 is recognized as the ‘guardian of the genome’, as its deficiency can lead to high frequency of cancer development in both mice and men due to loss of chromosome integrity [1]. The basal levels of p53 are held in check by binding to an E3 ligase Mdm2 (Hdm2 in humans) which promotes degradation of the protein [1]. In response to acute stress the interaction between Mdm2 and p53 decreases and levels of p53 are stabilized. Accumulation of p53 occurs predominantly in the nucleus where p53 binds to specific DNA sequences and alters the transcription of responsive genes [2]. Many p53 responsive genes are involved in the regulation of cell cycle, induction of apoptosis, or encode DNA repair enzymes. The altered activity of p53 responsive genes determines the outcome of the cell’s response to stress.

The death-inducing functions of p53 are tumour suppressive, due to its ability to induce apoptosis to eliminate damaged cells. Apoptosis is initiated by the permeabilization of the outer mitochondrial membrane, which is regulated by the members of BCL-2 family. The best characterized role of p53 in this context is as a nuclear transcription factor where it up-regulates the transcription of several pro-apoptotic members of the BCL-2 family, including BAX, PUMA, NOXA and BID, and inhibits the transcription of some anti-apoptotic members such as BCL-2 and BCL-X_L_ [3–6]. However, p53 can also induce apoptosis independently of its transcriptional function. Several apoptotic stimuli lead to the accumulation of p53 in the cytoplasm and the mitochondria are increasingly recognised as novel sites for this p53 activity. Although the complete physiological relevance of transcription-independent functions of p53 is not clear, they have been shown to deliver a decisive signal for apoptosis to commence [7, 8]. p53 has also been shown to function by direct interactions with, or activation of, BCL-2 family proteins at the mitochondria [9, 10].

Conventional cancer therapies, including genotoxic drugs and ionizing radiation, induce DNA damage and consequently activate and stabilize p53 (reviewed in [11]). The therapeutic value of p53 activation by such drugs is severely mitigated by p53’s role in causing the harmful side effects observed as a result of radiation and chemotherapy. Strategies that allow a short term blockage of p53 action in normal cells, while treating the p53 deficient tumors, therefore appear to be reasonably appealing in reducing the adverse effects of cancer therapies [1]. Two classes of small molecule inhibitors of p53 named pifithrin (PFT) have been identified, which target either the transcriptional or the mitochondrial activity of p53 [12, 13]. While PFT-α prevents p53 mediated transcriptional activation and subsequent apoptosis [12], PFT-µ, also known as 2-phenylethynesulfonamide or PES, and shown to have effects on HSP70 [14], was demonstrated to selectively inhibit the mitochondrial arm of p53 pathway by reducing the binding affinity of p53 to BCL-2 or BCL-X_L_ [13]. Both drugs can offer some protection against a lethal dose of ionizing radiation.

Etoposide is a widely used drug for chemotherapy that induces DNA damage by inhibition of Topoisomerase II [15]. Ensuing DNA damage response involves cell cycle arrest and DNA repair, but eventually cell death if repair is unsuccessful. Etoposide-treated cells accumulate at G2/M, which can occur in both p53-dependent and -independent pathways [16]. In the p53-independent pathway the cell cycle block induced by DNA damage is controlled by ATM/ATR protein kinases. The p53-dependent pathway on the other hand achieves the arrest at G2 by p53 mediated repression of cyclin B1/CDK1 promoters.

Despite the long history of studies investigating p53 and cell death by chemotherapy drugs such as etoposide, there remain a number of questions regarding the mechanism by which these processes are regulated. Previously, we found that etoposide can have differential effects on DNA damage response pathways when used at higher or lower concentrations [17]. The aim of this study was to probe the cellular and molecular events regulated by different concentrations of etoposide. We show that 1.5 µM of etoposide, a clinically relevant concentration shown to be achieved in the plasma of responders [18–20], clearly induces apoptosis through the transcription-independent p53 mitochondrial pathway. Even at higher concentrations of etoposide, where transcriptional activation via p53 occurs, our evidence suggests that the mitochondrial actions of p53 are more important for induction of cell death. These results may have implications in any attempts to modulate p53 function in the context of chemotherapy treatments.

## Results and discussion

### Etoposide increased the numbers of cells with sub-G1 DNA content in a concentration-dependent manner

**Fig. 1:**
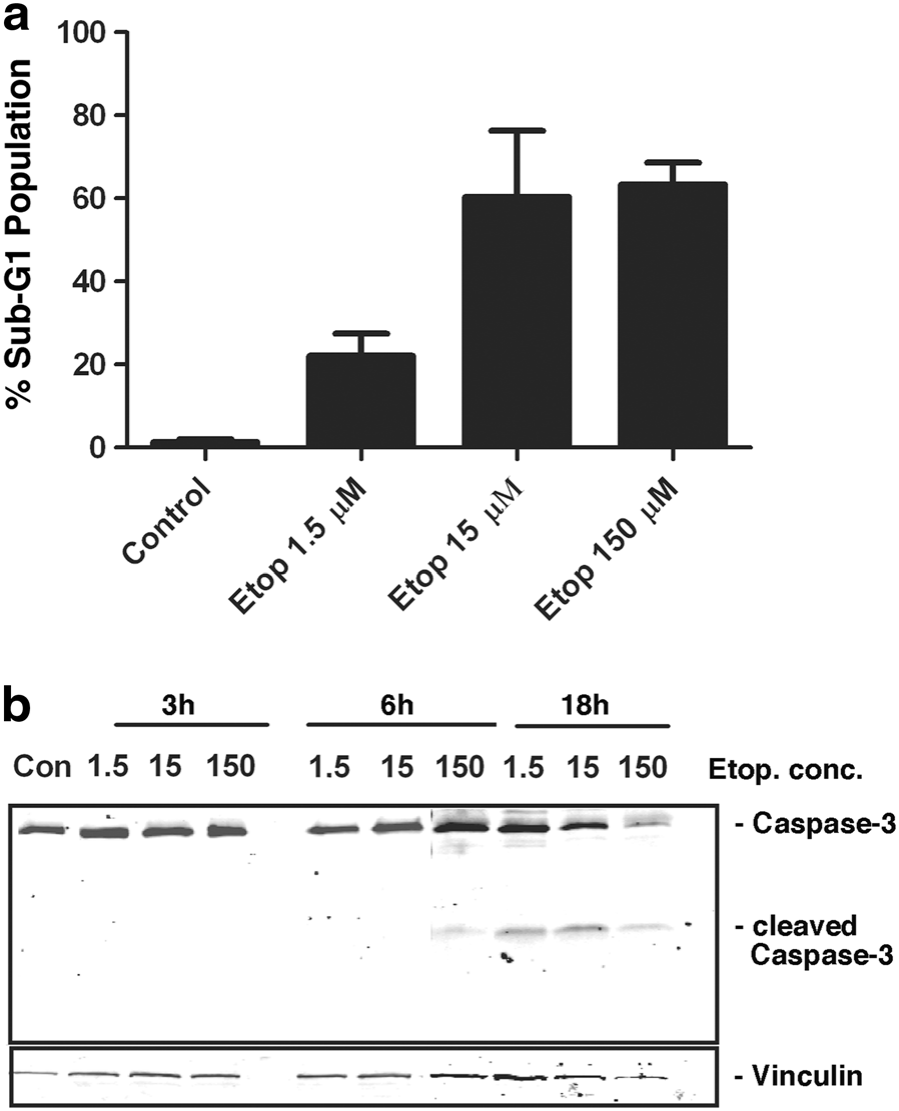
Concentration-dependent etoposide-induced apoptosis. **a** Flow cytometry analysis of MEFs was performed to determine the percentage of cells with DNA content below the threshold for cells in G1, following 18 h treatment with 1.5, 15 or 150 µM etoposide. Data shown are mean + SD from seven independent experiments with three replicates each. **b** MEFs were treated with 1.5, 15 or 150 µM etoposide for 3, 6 or 18 h. Con indicates extract from untreated cells. Total cell proteins were probed with anti-Caspase-3 antibody. Vinculin was used as the loading control. Figures are representative of three independent experiments showing similar results.

In our previous studies we have investigated the role of MCL-1 in DNA damage response by using a low concentration of etoposide [17, 21]. To further our understanding of the differences in the cellular responses to treatment with low compared to high concentrations of etoposide, mouse embryo fibroblasts (MEFs) were treated with etoposide at different concentrations and analyzed for their apoptotic response after 18 h. Treatment of MEFs with etoposide induced cell death, which was verified by the appearance of sub-diploid G1 peaks in flow cytometry analysis of permeabilized cells stained with propidium iodide. As shown in Fig. 1a, approximately 22% cells underwent apoptosis following treatment with 1.5 µM etoposide for 18 h, compared to 60% with 15 µM and 65% with 150 µM etoposide. A representative experiment showing the raw data from flow cytometry is shown in Additional file 1: Figure S1.

We next investigated the activation of caspase-3 in response to different concentrations of etoposide. Western blot analysis with caspase-3 antibody showed that 150 µM of etoposide induces robust cleavage of caspase-3 within 6 h while 1.5 or 15 µM activate caspase-3 only after 18 h (Fig. 1b). These results confirm that a low concentration of etoposide (1.5 µM) is indeed able to induce apoptosis in MEFs following treatment for less than 24 h. Thus, these conditions were subsequently used to compare effects of etoposide in inducing measurable cell death, allowing us to more carefully query any differences in the molecular responses to the various concentrations of drug.

### Low or high concentrations of etoposide have different effects on transcriptional regulation by p53

**Fig. 2:**
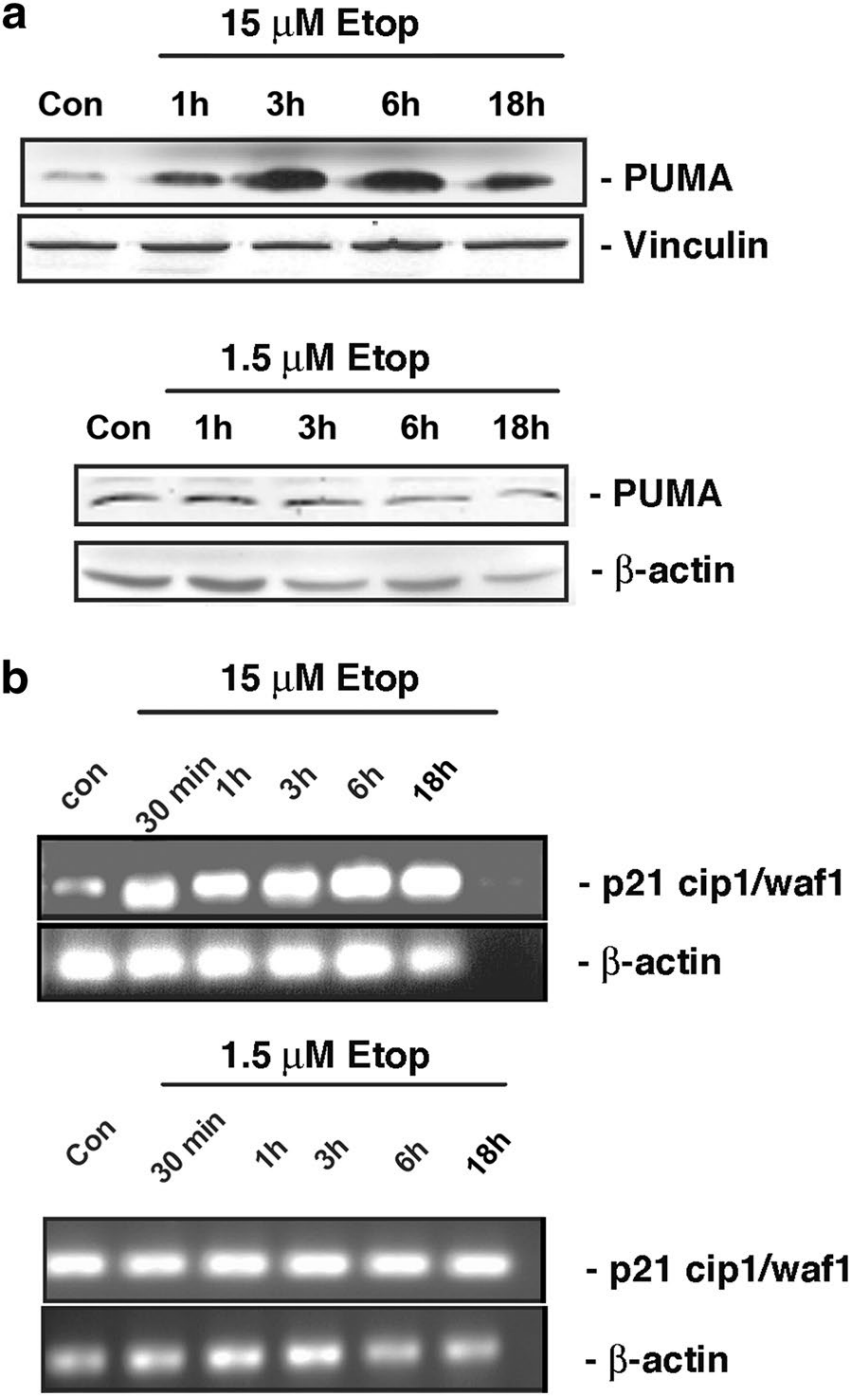
Etoposide effect on p53-mediated transcriptional events. **a** MEFs were treated with 15 µM of etoposide (*upper panel*) or 1.5 µM of etoposide (*lower panel*) for 1, 3, 6 or 18 h. Total cell proteins were probed with anti-PUMA antibody. Vinculin or actin was used as the loading control. **b** MEFS were treated with 15 µM of etoposide (*upper panel*) or 1.5 µM of etoposide (*lower panel*) for 30 min, 1, 3, 6 or 18 h. Total RNA was isolated and reverse transcribed into cDNA. The expression of p21^CIP1/WAF1^ was determined by RT-PCR using specific primers. β-actin was used a loading control. Figures are representative of three independent experiments.

Tumor suppressor protein p53 plays an important role in DNA damage-induced apoptosis, at least partly by acting as a transcription factor to direct the expression of apoptotic mediators. We investigated the effect of etoposide on two representative transcriptional targets of p53, the BH3-only pro-apoptotic protein PUMA and cell cycle inhibitor p21^CIP1/WAF1^. Our results showed that treatment of cells with 15 µM of etoposide induced up-regulation of PUMA protein expression (known to be directly correlated with its p53-dependent transcription) within 1 h, which increased further at 3 and 6 h, before declining after 18 h (Fig. 2a, upper panel). A similar increase was observed in p21^CIP1/WAF1^ mRNA levels, which showed an increase at 30 min and remained elevated even after 18 h incubation (Fig. 2b, upper panel). When cells were treated similarly with 1.5 µM etoposide, which can still induce substantial cell killing, no increase in the expression of either PUMA protein or p21^CIP1/WAF1^ mRNA levels was observed over the same time periods (Fig. 2a, b, lower panels). These results suggested that the cell death observed in response to treatment with 1.5 µM etoposide was independent of these two transcriptional events mediated by p53.

### PFT-α failed to rescue the cells from etoposide-induced apoptosis

**Fig. 3:**
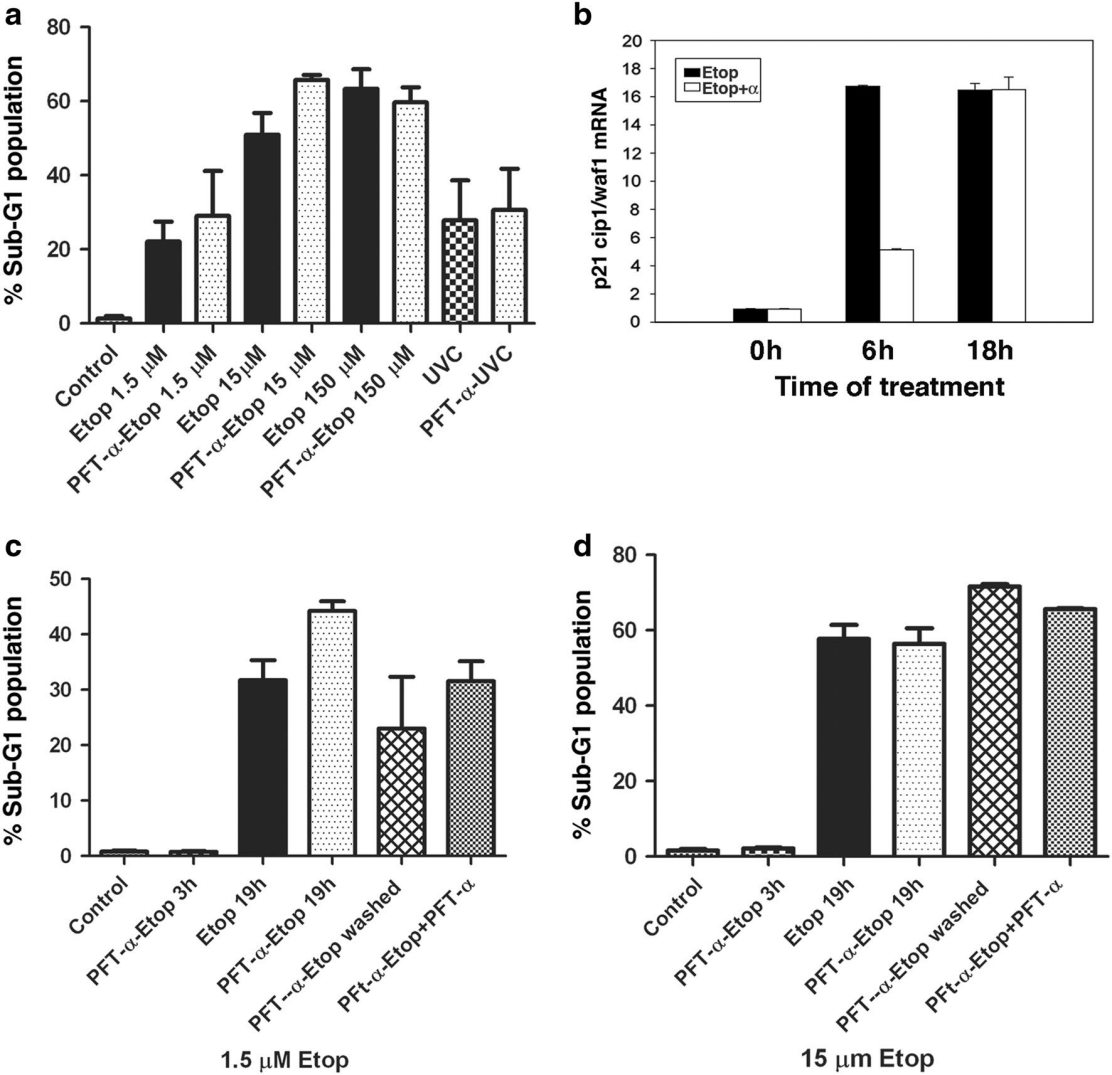
Effect of PFT-α on etoposide-induced apoptosis. **a** MEFs, pre-treated with 30 μM PFT-α followed by 18 h treatment with 1.5, 15 or 150 µM etoposide, were analyzed by flow cytometry to determine percentage of cells having sub-G1 DNA content. Cells were treated in parallel with UVC and analyzed after 18 h. Control indicates normally proliferating cells. Columns represent percentage of cells having sub-G1 DNA content, as analyzed by flow cytometry. Data are mean + SD of three independent experiments with three replicates each. **b** mRNA was extracted from untreated (0 h), 6 and 18 h treatment with 15 µM of etoposide (*black bars*) or pre-treatment with PFT-α followed by etoposide (*open bars*). The relative expression of p21^cip1/waf1^ was determined by qRT-PCR (mean ± SD from three independent experiments). The values of p21^cip1/waf1^ were normalized to β-actin. **c**, **d** Parallel studies used 1.5 or 15 µM etoposide. Sub-G1 population of MEFs was measured as in A for cells that were untreated (Control), pre-treated with 30 μM PFT-α followed by etoposide for 3 h (PFT-α-Etop 3 h), etoposide alone for 19 h (Etop 19 h), pre-treated with PFT-α followed by etoposide for 19 h (PFT-α-Etop 19 h), washed after 3 h of co-treatment, followed by further incubation for 18 h (PFT-α-Etop washed) and washed after 3 h, and PFT-α was added back for 18 h (PFT-α-Etop + PFT-α). Columns represent percentage of cells having sub-G1 DNA content; representative of three experiments with similar results.

To determine whether higher concentrations of etoposide may be inducing apoptosis through the transcription-dependent actions of p53, we used the small molecule inhibitor PFT-α. PFT-α has been shown to interfere with the expression of p53-inducible genes [12, 22, 23]. Although originally identified as a selective inhibitor of p53-induced transcription, it is now known to have other p53 independent functions [24]. Pre-treatment of cells with 30 µM PFT-α, followed by 18 h treatment with either 1.5 or 15 or 150 µM of etoposide failed to rescue the cells from DNA damage-induced apoptosis (Fig. 3a). In parallel experiments, cells were pre-treated with PFT-α and subsequently exposed to UVC radiation to induce DNA damage mediated apoptosis. PFT-α similarly failed to exert any effect on UV treatment–induced apoptosis.

The inability of PFT-α to rescue cells treated with any concentration of etoposide or exposure to UV radiation was intriguing. We therefore confirmed the efficacy of PFT-α in inhibiting the transcriptional up-regulation of p21^CIP1/WAF1^ gene expression. MEFs were pre-treated with PFT-α followed by addition of 15 µM etoposide for various time periods. PFT-α effectively inhibited the up-regulation in p21^CIP1/WAF1^ for up to 6 h (Fig. 3b), suggesting that the drug was indeed effective in suppressing p53-regulated transcription. However, it failed to do so in cells that were treated for 18 h, a result that may be explained based on studies showing that the half-life of PFT-α is approximately 5 h in physiological conditions [25]. We can conclude that blocking p53-dependent transcriptional events does not affect etoposide-induced cell death.

In another experimental approach, cells were incubated with PFT-α and subsequently treated with etoposide for 18 h, or treated with PFT-α and etoposide for 3 h only, and the drug washed out prior to incubation in the presence or absence of PFT-α for 18 h. Cell death was assessed by measurement of sub-G1 DNA content. While no death is detected after 3 h of etoposide treatment (not shown), 3 h treatment with either 1.5 or 15 μM etoposide, followed by washing and further incubation, was sufficient to induce cell death, almost as well as continual exposure to etoposide. However, this was not affected by the presence of PFT-α, despite its ability to block p53-mediated transcription (Fig. 3b–d). Together, these experiments support the conclusion that etoposide-induced cell death is not due to an effect on p53-dependent transcription.

### PES inhibits etoposide–induced apoptosis and cell cycle checkpoint response

**Fig. 4:**
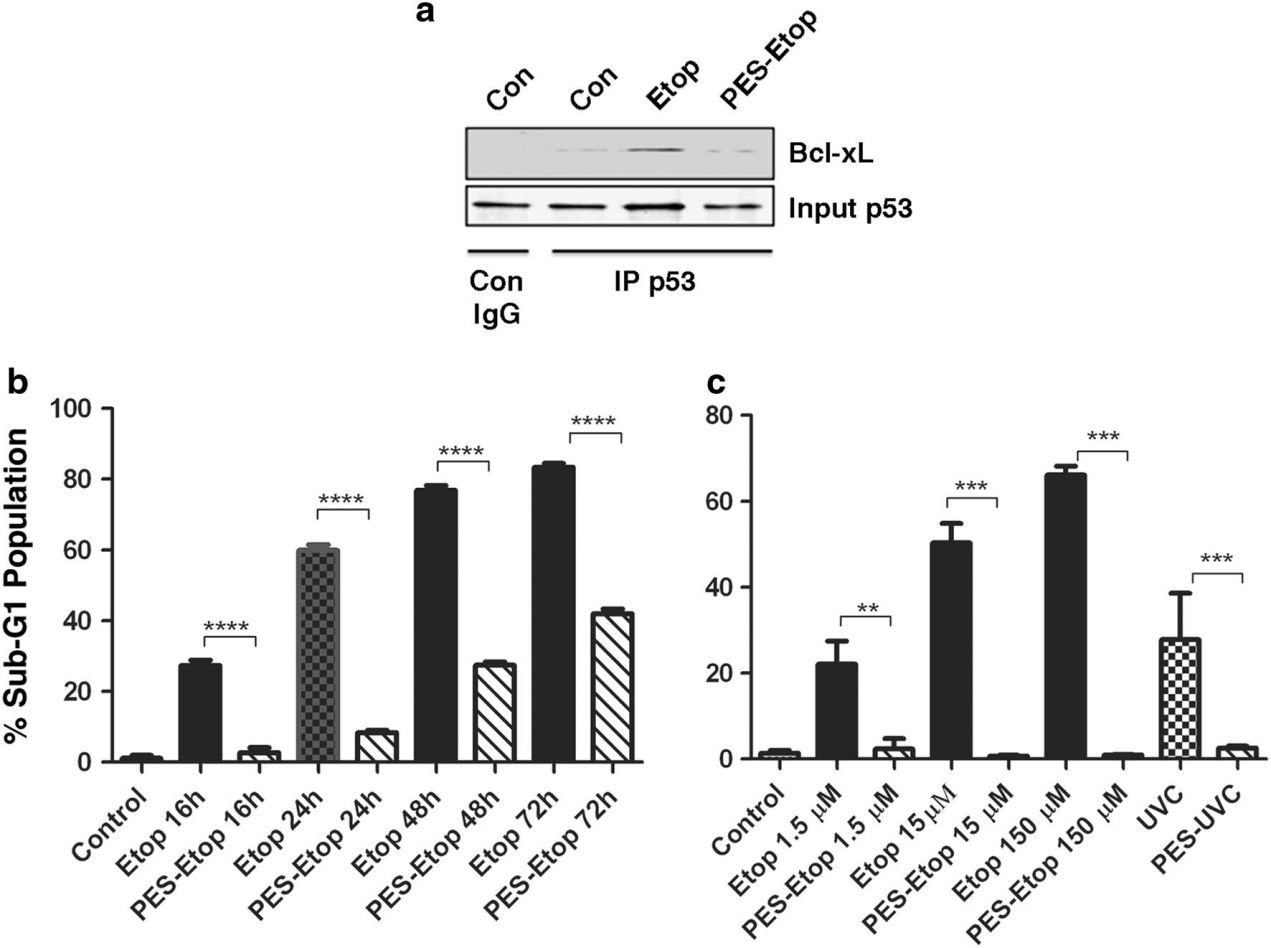
Effect of PES on etoposide-induced cell death. **a** MEFs were either untreated (Con) or treated with etoposide (Etop) for 6 h, or pre-treated with 10 μM of PES followed by 1.5 μM of etoposide for 6 h. Mitochondrial extracts were immunoprecipitated with anti-p53 antibody and probed for Bcl-xL. The *first lane* (IP Con IgG) represents mitochondrial extracts from untreated (Con) cells immunoprecipitated with rabbit IgG alone Input p53 represents the starting material unbound to the Agarose G beads, probed for the presence of p53. **b** MEFs were treated with either 1.5 µM etoposide alone or pre-treated with 10 μM of PES and followed by 1.5 µM etoposide for various times. Columns represent percentage of cells having sub-G1 DNA content. A one-way ANOVA was carried out to compare the treatment groups. Post hoc comparison using the Tukey HSD test indicated significant inhibitory effect of PES treatment on the percentage of cells having sub-G1 DNA content. Data shown are mean + SD from 3 independent experiments with three replicates each. **c** Analysis of MEFs was done as in B; cells were pre-treated with 10 μM PES followed by 18 h treatment with 1.5, 15 or 150 µM etoposide. Cells were treated in parallel with UVC and followed for 18 h. Statistical analysis was as in **b**. Data are mean + SD of three repeat analyses.

Since we had shown that etoposide-induced cell death is independent of p53’s transcriptional regulation, we tested the effect of pre-treatment with 10 µM PES. PES is a small molecule that prevents the association of p53 to the mitochondria [13, 26]. Western blot analysis of mitochondrial extracts following etoposide treatment showed a dramatic increase in mitochondrial p53 abundance, which was blocked in PES-treated cells (data not shown). To directly assess the effect of PES on p53’s interaction with a known binding partner at the mitochondria, p53 was immunoprecipitated and the immunoprecipitate was probed for BCL-xL levels. As shown in Fig. 4a, PES disrupted the p53/BCL-xL interaction. It should be noted that PES had little to no effect on total cellular p53 levels (see other data below). Furthermore, pre-treatment of MEFs with PES dramatically inhibited etoposide-induced generation of sub-G1 DNA content, particularly at earlier times (Fig. 4b). Moreover, PES was able to effectively block death of cells that were treated with a 10–100 fold higher concentration of etoposide over a period of 18 h, as well as cell death induced by UVC treatment (Fig. 4c). Together, this provides further evidence that the primary means by which etoposide induces cell death is via the transcription-independent functions of p53.

The transcription independent death pathway of p53 has been suggested to occur via several possible pathways: p53 may act as a ‘super’ BH3-only protein and may interact with the multi domain anti-apoptotic BCL-2 family members to liberate pro-apoptotic members from inhibitory complexes [27–29] or p53 can interact directly with pro-apoptotic BAK to release cytochrome C [29]. Yet another model proposed that stress-induced cytosolic p53 is sequestered by soluble anti-apoptotic BCL-X_L_ and transcriptional activation of PUMA displaces p53, which then activates monomeric cytosolic BAX to induce apoptosis [10]. Our results showing that activation of PUMA was not required are not consistent with the latter possibility, and likely support a role for p53 as a BH3-only protein at the mitochondria.

**Fig. 5:**
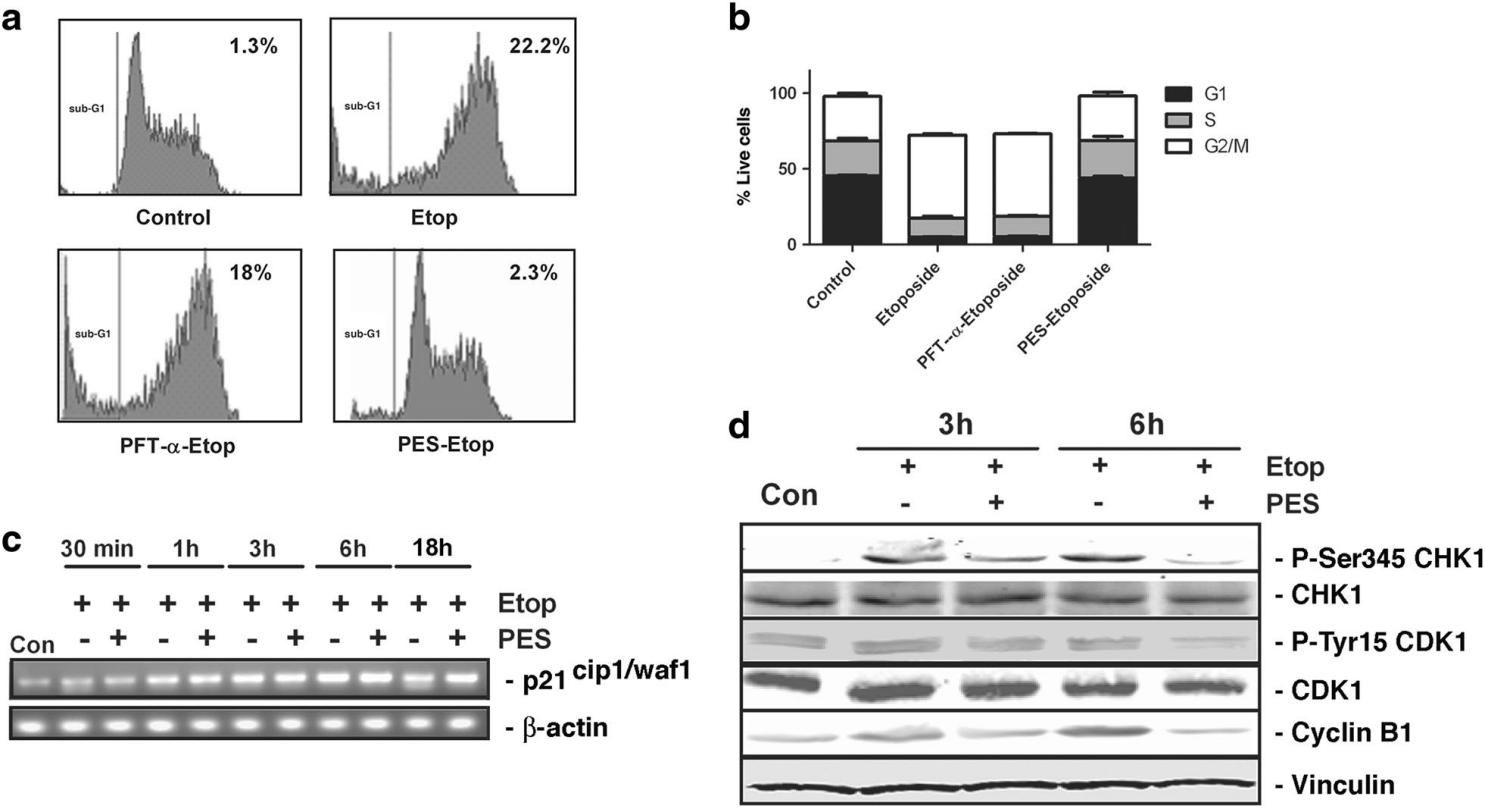
Effect of PES on cell cycle events. **a** Representative flow cytometry profiles of MEFs, with control (no treatment), treatment with 1.5 µM etoposide for 18 h, pre-treatment with 30 μM of PFT-α followed by 18 h treatment with 1.5 µM etoposide or pre-treatment with 10 μM of PES followed by 18 h treatment with 1.5 µM etoposide. Percentage of cells having sub-G1 DNA content is indicated. Data shown are representative of six experiments with similar results. **b** *Columns* represent distribution of cells in G1, S and G2/M phases of cell cycle, as analyzed by flow cytometry. Data are mean + SD of three independent experiments with three replicates each. **c** MEFs were treated for various times with 15 µM of etoposide either alone or pre-treated with 10 μM of PES. Total RNA was isolated and reverse transcribed into cDNA. The expression of p21^cip1/waf1^ was determined using specific primers in RT-PCR. β-actin was used as a loading control. Representative of three independent experiments. **d** MEFs were untreated (Con) or treated with 1.5 µM of etoposide for 3 or 6 h alone or pre-treated with 10 μM PES. Total cell proteins were probed with anti-Ser345 CHK1, anti-CHK1, anti-phospho-Tyr 15 CDK1, anti-CDK1, Cyclin B1 antibodies, and anti-vinculin as the loading control.

We next investigated the cell cycle status of the MEFs in the various treatment conditions. Our results showed that in response to treatment with etoposide, MEFs undergo a DNA damage-induced arrest at the G2/M phase of the cell cycle and apoptotic cells with sub-G1 levels of DNA can be detected, as expected (Fig. 5a). Interestingly, while PFT-α had no effect, treatment of cells with PES overrides the etoposide-induced DNA damage checkpoint at G2/M (Fig. 5a, b). Similar results were obtained when HeLa cells were pre-treated with PES and followed by treatment with etoposide (data not shown). Etoposide is known to activate checkpoint response, which delays the progression through the cell cycle. Since the progression of cell cycle from G2 to mitosis is driven by cyclin dependent kinase CDK1 [30], it is a prime target of DNA damage response proteins for instigation of G2/M arrest. The CDK inhibitor p21^CIP1/WAF1^ has been shown to be up-regulated at both G1/S and G2/M checkpoints and studies of Ding et al. have reported an increase in its expression in response to etoposide treatment in a p53-dependent manner [31]. We therefore determined whether the ability of PES to alter cell cycle effects of etoposide is due to any effect on down-regulation of p21^CIP1/WAF1^ expression. As seen in Fig. 5c, PES treatment had no apparent effect on etoposide-induced p21^CIP1/WAF1^ transcription. Again, this finding supports the suggestion that etoposide’s effects are mediated largely through p53’s functions at the mitochondria, unrelated to transcriptional regulation.

We next investigated the effect of PES treatment on CDK1/Cyclin B1 activity. Phosphorylation of CDK1 at the inhibitory site, Tyr15, is a key event controlling the G2/M switch. MEFs were treated with either etoposide alone or in combination with PES for 3 or 6 h and expression of phospho-Tyr15-CDK1 was investigated by immunoblotting. As shown in Fig. 5d, an increase in phospho-Tyr15-CDK1 was observed at 3 and 6 h post treatment with etoposide, as expected in cells blocked at G2/M. Pre-treatment with PES decreased the etoposide-induced CDK1 phosphorylation at both 3 and 6 h. We next examined the effect of PES on Cyclin B1 expression, which is an absolute requirement for CDK1 activity. Similar to phospho-Tyr15-CDK1, an increase in Cyclin B1 expression was observed at 3 and 6 h post-etoposide treatment. Interestingly, the Cyclin B1 level was much lower in the presence of PES. A decrease in phospho-Tyr15-CDK1/Cyclin B1 expression is consistent with cells escaping the G2 checkpoint arrest that normally occurs in response to etoposide treatment. Activation of Checkpoint kinase 1 (CHK1) blocks the entry into mitosis by phosphorylating members of CDC25 family of phosphatases, which activate cyclin B1-CDK1 through dephosphorylation of Thr14 and Tyr15 [32, 33]. Hence, we next examined whether the inhibitory effect of PES on Tyr15-CDK1 was the consequence of CHK1 inhibition. We probed the same membrane with anti-phospho-Ser345 CHK1 antibody. Our results showed that, as expected, CHK1 was activated in response to etoposide treatment at 3 and 6 h. Pre-treatment with PES resulted in a marked reduction in CHK1 activation which would eventually impede the cell cycle arrest by allowing the cells to enter mitosis. These results highlight several possible mechanisms by which PES treatment is overriding the etoposide-induced cell cycle arrest.

Treatment with etoposide causes G2 arrest by p53-dependent and independent pathways and either pathway can adequately cause G2 arrest [16]. The p53-dependent pathway can exert its inhibitory effects on cell cycle progression through either direct binding of p21^cip1/waf1^ to CDK1 [34], down-regulation of CDK1/Cyclin B1 protein levels [16] or p21^cip1/waf1^ mediated prevention of inhibitory phosphorylation of P130 and P107 by CDKs, which in turn represses the transcription of several genes required for progression through G2/M [35]. The p53-independent pathway on the other hand is regulated by DNA damage response kinases ATM and ATR [36]. Our results showed that in response to treatment with 1.5 µM of etoposide, the cells arrest at G2 through the p53-independent pathway and that PES is able to bypass it by inhibiting the phosphorylation of CHK1 on Ser 345 (Fig. 5d). These data contradict the findings of Balaburski et al. who showed that PES treatment leads to G2/M arrest by inhibiting the activity of Anaphase promoting complex/cyclosome and thus prevents the degradation of Cyclin B1 [37]. The reason for this apparent discrepancy is not clear as Balaburski et al. used HeLa cells in their studies and we have also confirmed that our results, first observed in MEFs, are also observed in HeLa cells.

### PES enhances deacetylation of Lys373/382 of p53

**Fig. 6:**
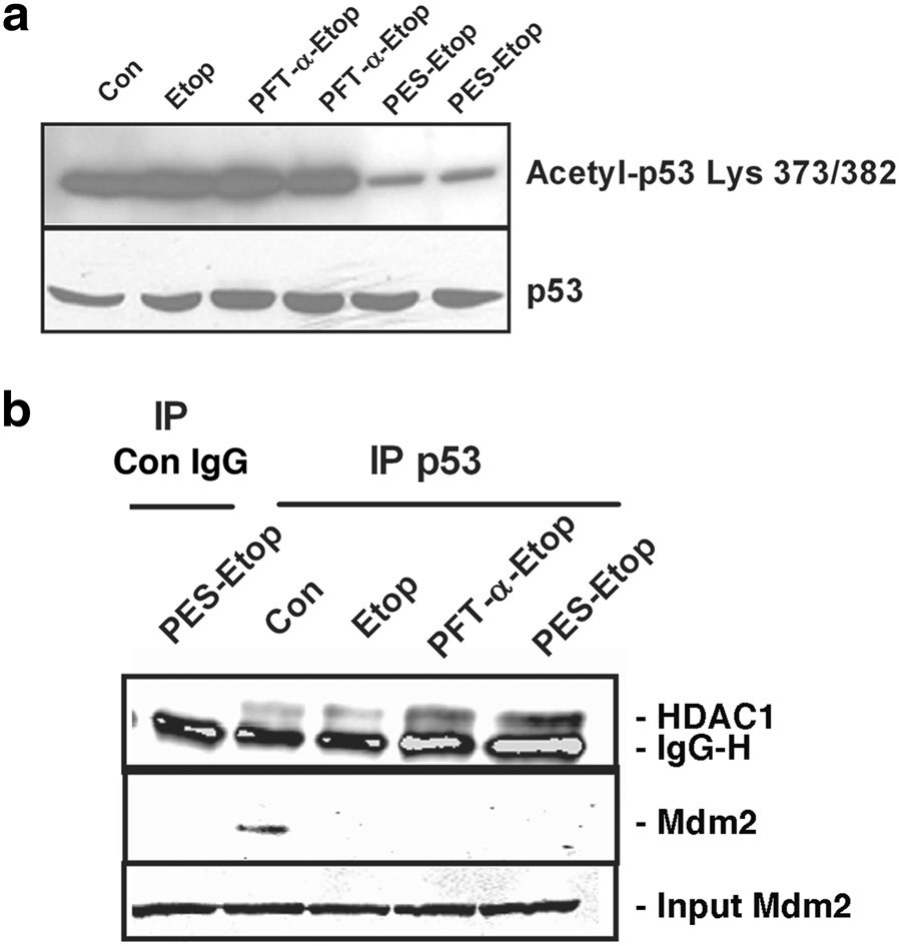
Effect of drugs on p53 acetylation and complex formation. **a** MEFs were either left untreated (Con) or treated with etoposide (Etop) alone or in combination with either PFT-α or PES for 3 h. Total cell extracts were prepared. The blot was probed with anti-Lys373/382 p53 antibody or anti-p53 as a loading control. **b** MEFs were either untreated (Con) or treated with etoposide (Etop) for 3 h, or pre-treated with either 30 µM of PFT-α or 10 μM of PES followed by 1.5 μM of etoposide for 3 h. Total cell extracts were immunoprecipitated with anti-p53 antibody and probed for HDAC1 and Mdm2. The first lane (IP Con IgG) represents PES and etoposide-treated extracts immunoprecipitated with rabbit IgG alone to indicate position of the IgG heavy chain (IgGH). Input Mdm2 represents the starting material unbound to the Agarose G beads, probed for the presence of Mdm2.

The stability and activity of p53 is regulated by post-translational modifications [38]. In particular, acetylation of lysine residues in the C-terminal regulatory domain of p53 has been shown to correlate well with the stability and activity of p53 [39]. We examined the effect of PES on the acetylation of Lys 373/382 of p53. Since MEFs used in the study were transformed using SV40 large T antigen, the p53 is constitutively acetylated at Lys 373/382. Interestingly when cells were treated with either PFT-α or PES alone, a decrease in Lys 373/382 acetylation of p53 was observed (data not shown). However, treatment of MEFs with PFT-α was unable to exert any effect on p53 acetylation when added with etoposide, while PES added in the presence of etoposide resulted in reduced acetylation on residues 373 and 382 (Fig. 6a).

### PES mediated deacetylation of p53 is not regulated by Mdm2-HDAC1

Mdm2 has been shown to negatively regulate acetylation of p53 [38, 40]. The effect of Mdm2 on p53 acetylation was reported to result following recruitment of a complex containing Histone deacetylase 1 (HDAC1) [41]. We therefore sought to determine whether p53 in normally proliferating MEFs was bound to Mdm2 and whether treatment with PES would enhance recruitment of Mdm2-HDAC1 complex. We used co-immunoprecipitation studies to determine these interactions. Our results showed that in untreated MEF cells, p53 interacts with both endogenous Mdm2 as well as HDAC1. However, pre-treatment of cells with either PFT-α or PES resulted in an increase in Mdm2-independent p53/HDAC1 association. We did not observe an effect on the HDAC1 association upon treatment with etoposide alone. It is noteworthy that despite recruitment of HDAC1 to p53 in PFT-α treated MEFs, no deacetylation of p53 on Lys373/382 is observed in cells co-treated with etoposide (Fig. 6a, b) suggesting that presence of HDAC1 alone may not be sufficient for deacetylation.

p53 is transiently activated and stabilized in response to various stimuli by post-translational modifications. These modifications include phosphorylation [42], which has been shown to interfere with the ability of Mdm2 to negatively regulate p53 [43] and acetylation, which has been shown to promote p53 stability [41]. It is well established that Mdm2 ubiquitinates p53 on lysine residues 373/382 and hence acetylation of these residues prevents the ubiquitin mediated turnover of p53 [38, 42]. Therefore, our finding that p53 is associated with Mdm2 and HDAC1 in MEFs that normally express p53 acetylated on Lysine 373/382 residues are intriguing. One explanation for these observations could be that the presence of another protein partner is required in the complex for efficient deacetylation. The pre-treatment with either PFT-α or PES, which affect p53 very differently, both caused increased recruitment of HDAC1 to p53. In this context it should be mentioned that recruitment of HDAC1 is Mdm2 independent and while PES causes deacetylation in the presence or absence of etoposide treatment, PFT-α deacetylates only when added alone. Etoposide and small molecule inhibitors PFT-α and PES have distinct activities related to their effects on p53 and perhaps the composition of the complex formed is a reflection of that. These observations are indeed intriguing and necessitate further investigation.

## Conclusion

Despite decades of study in numerous experimental systems, the pathways by which p53 is regulated following treatment with etoposide, a commonly used drug for chemotherapy and a potent inducer of DNA damage, remain incompletely characterized. In this study, we asked whether etoposide induced cell death via p53 transcription-dependent events, or whether the mitochondrial events targeted by p53 were involved. Our first hint that transcription-dependent regulation by p53 is not required came from observations in which lower concentrations of etoposide were used. While the low concentration of etoposide was able to induce substantial cell death, it had no detectable effect on p53-dependent transcription. We next used known inhibitors of p53, PFT-α and PES, which target p53 transcriptional and mitochondrial functions, respectively. These compounds have both been reported to suppress death in mice exposed to radiation, and could thus be useful to block the harmful effects of chemotherapy that occur via p53-induced death. In our studies, PFT-α was able to suppress p53-mediated transcription induced by etoposide treatment. However, it was unable to suppress cell death induced by either low or high concentrations of etoposide. These results suggest that PFT-α cannot effectively block etoposide-induced death, despite blocking the induction of transcription via p53. On the other hand, PES was very effective in blocking etoposide-induced cell death, as determined by its ability to block the appearance of sub-G1 DNA content. We showed that PES may act in multiple ways to bypass the effects of etoposide, including (1) a dramatic effect in blocking etoposide-induced association of p53 with the mitochondria (without any major effect on p53 total expression, (2) blocking the G2/M arrest induced by etoposide, likely via effects on Cyclin B1/CDK1, and (3) blocking acetylation of p53.

The in vivo relevance of the mitochondrial effects of p53 is emphasized by the observation that within 30 min of radiation or injection of a clinical dose of etoposide in drug sensitive organs, activation of caspase-3 is observed, and this precedes detectable p53 dependent gene activation [44]. The significance of this observation has been underscored further by our observation that cell death still ensues when p53-dependent transcription is blocked for the duration of DNA damage. Based on these observations and those of others [45], we would like to stress that the p53 mitochondrial pathway is pivotal in exerting the lethal effects of chemo-/radiotherapy observed in sensitive cells.

In conclusion, our observation that a low, but clinically pertinent, concentration of etoposide is able to elicit death of cells whose p53 transcription function has been blocked has implications for use of etoposide in combination with chemotherapeutic agents that block transcription (reviewed by Ljungman and Lane [46]) or in tumors with mutations in the p53 DNA-binding core domain. To provide a fail-safe mechanism of eliminating cancerous cells, efforts could focus on enhancing the effects of p53 in causing cell death via its effects at the mitochondria. At the very least, our work supports effects to combine etoposide treatment in p53-inactive tumours, with agents that induce apoptosis via mitochondrial pathways dependent on BCL-2 family members.

## Methods

### Cell lines

Wild type MEFs (a kind gift from Dr. J. Opferman) were obtained from mixed 129/B6 background embryos harvested on E10.5. The primary P3 cells were immortalized by SV40 and cloned. Single cell clones were cultured in DMEM supplemented with 10% fetal bovine serum.

### Antibodies and reagents

Normal rabbit IgG and anti-actin (I-19) were purchased from Santa Cruz Biotechnology (Santa Cruz, CA, USA). Monoclonal anti-human vinculin was purchased from Sigma-Aldrich (Oakville, ON, USA). Rabbit anti-phospho-Tyr15 CDK1, rabbit anti-phospho-Ser345 CHK1, Bcl-xL and anti-caspase-3 antibodies were from Cell Signaling Technology (Beverly, MA, USA). Anti-CHK1 (D-7) and anti-cyclin B1 (H 433) were purchased from Santa Cruz Biotechnology (Santa Cruz, CA, USA). Rabbit polyclonal p53 antibody (CM5) was from Leica Microsystems (Concord, ON, USA), anti-acetyl-p53 (Lys 373, Lys 382) was from Millipore (Billerica, MA, USA) and anti-COX IV was from Abcam (Toronto, ON, USA). Etoposide, PFT-α and PES were purchased from Calbiochem (La Jolla, CA, USA). Fetal bovine serum and Protein-G Agarose beads were purchased from Invitrogen (Carlsbad, CA, USA).

### Cell treatments

The stock solutions of etoposide, PFT-α and PES were prepared in DMSO. An equal amount of DMSO was added to the control cells in each experiment. MEFs were treated with 1.5, 15 or 150 µM of etoposide for indicated periods of time. Pre-treatments with PFT-α or PES were carried out by incubation of cells with either 30 µM of PFT-α or 10 µM of PES for 10 min prior to the addition of etoposide. The UV irradiation was performed by removal of medium, washing once with PBS and exposure to a controlled dose of UVC (254 nm) light using an UltraLum cross-linker (Claremont, CA, USA).

### Immunoblotting

Cells were washed with PBS and suspended in ice-cold solubilization buffer (20 mM Tris HCL pH 8.0, 1% NP40, 10% glycerol, 137 mM NaCl, 10 mM NaF, with Roche protease inhibitor cocktail) then sonicated for 5 s before centrifugation at 32,000×*g* for 5 min. Equivalent concentrations of protein were resolved using SDS–polyacrylamide gel electrophoresis and transferred to nitrocellulose membranes. Membranes were blocked for 1 h in TBST-5% low-fat milk followed by overnight incubation at 4°C with appropriate antibody and detection of IR-conjugated secondary antibodies using a LiCor Odyssey. The mitochondrial extracts were prepared by using mitochondria isolation kit for cultured cells (Thermo Scientific) according to the manufacturer’s recommendations.

### Immunoprecipitation

For immunoprecipitation of p53 complexes, total cell extracts were pre-cleared with 20 μl of Protein G agarose beads for 30 min. One μg/ml anti-p53, or normal rabbit IgG antibody was added and after an overnight incubation, the immunoprecipitates were collected by adding 50 μl of Protein G agarose beads. Beads were washed four times with solubilization buffer.

### RT-PCR and quantitative real-time PCR

Total RNA was isolated from these cells using the GeneJet RNA purification kit (Thermo Scientific) according to the manufacturer’s recommendations. Total RNA was reverse transcribed into cDNA using the RevertAid H minus reverse transcriptase (Thermo Scientific). The expression of p21^CIP1/WAF1^ was determined by RT-PCR using specific primers (sequence described below). β-actin was used a loading control.

For real-time PCR, amplification was performed using LightCyclerFastStart DNA Master Plus SYBR (Roche Applied Science, Mannheim, Germany) and using the comparative cycle threshold method (2^−∆∆CT^) to quantify gene expression. The mRNA levels were normalized to mouse β-actin expression (sense primer: 5′-GAGCACAGCTTCTTTGCAGCT-3′ and antisense primer: 5′-CCCACATAGGAGTCCTTCTAGCC-3′). The primer sequences for the p21 were 5′-GTGTGCCGTTGTCTCTTCGG-3′ and 5′-CTCAGGTAGACCTTGGGCAG-3′.

### Flow cytometry for cell cycle and subdiploid DNA staining

Cells for flow cytometric analysis were fixed in 70% (v/v) ethanol. The cells were subsequently stained in PBS containing 50 μg/ml of PI (propidium iodide), 100 μg of RNase A and 0.1% glucose. Cells were analysed using BD FACSCanto II (BD Biosciences, San Jose, CA, USA).

## Additional file

Additional file 1:
**Figure S1.** Etoposide-induced apoptosis is concentration dependent. Representative flow cytometry analysis of control cells compared to treatment with various concentrations of etoposide. Cells were fixed and stained with propidium iodide as described. Cells with sub-G1 DNA content, or in G1, S and G2 phases of the cell cycle are indicated.

## Abbreviations

CDK1: cyclin dependent kinase 1
CHK1: checkpoint kinase 1
HDAC1: histone deacetylase 1
MEF: mouse embryo fibroblast
PFT-α: pifithrin-α
PES: 2-phenylethynesulfonamide
UVC: ultraviolet-C (short wavelength)
