# Supplementary figures and images for: Etoposide induces cell death via mitochondrial-dependent actions of p53
Source: Cancer Cell Int. 2015 Aug 7;15:79. doi: 10.1186/s12935-015-0231-z (PMC4527242; doi:10.1186/s12935-015-0231-z)

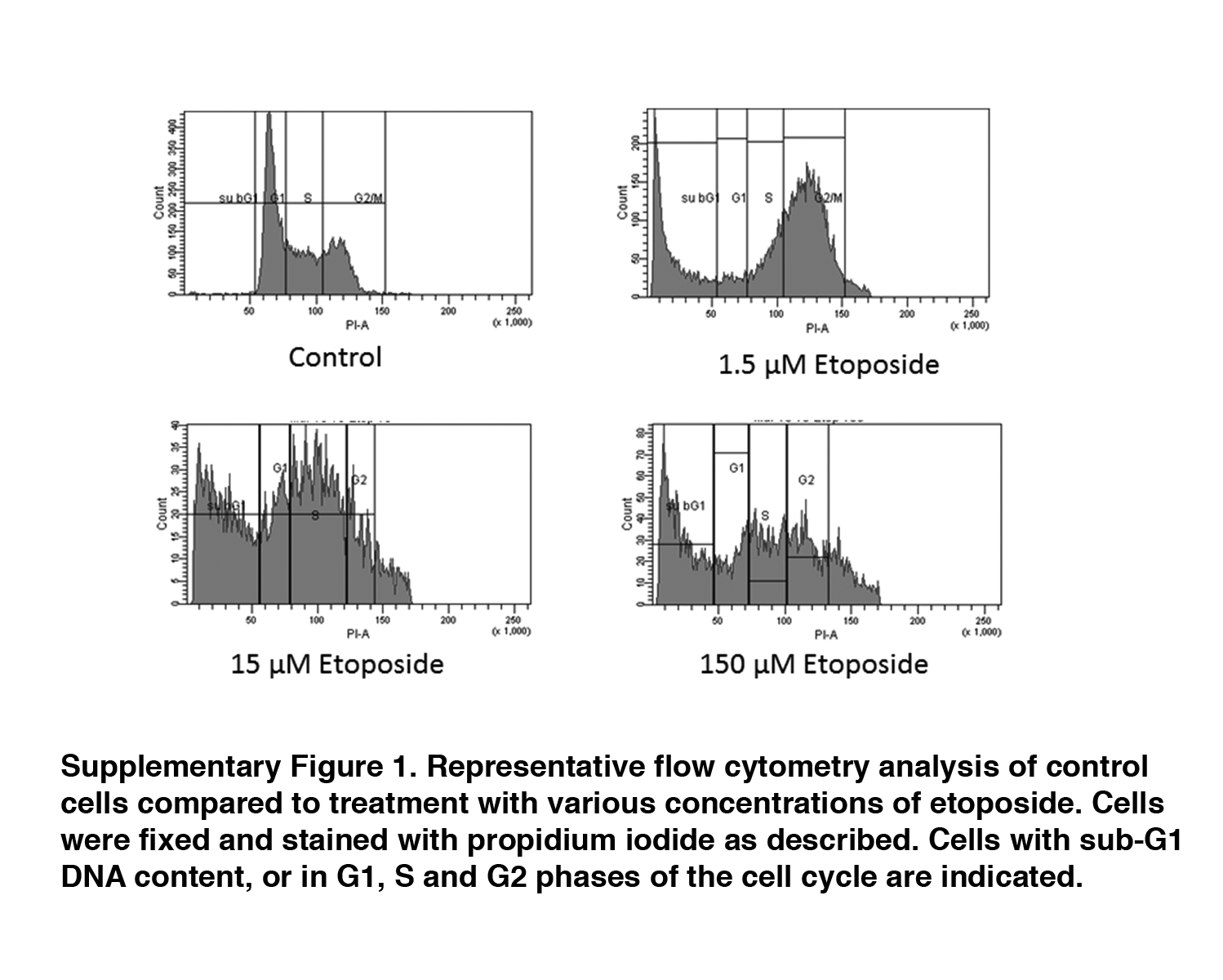

Supplement: Additional file 1: — Figure S1. Etoposide-induced apoptosis is concentration dependent. Representative flow cytometry analysis of control cells compared to treatment with various concentrations of etoposide. Cells were fixed and stained with propidium iodide as described. Cells with sub-G1 DNA content, or in G1, S and G2 phases of the cell cycle are indicated. [file 12935_2015_231_MOESM1_ESM.tif]
